# 응급실에 내원하는 국내 암 환자의 특성과 체류시간 및 72시간 이내 재방문: 후향적 서술적 조사연구

**DOI:** 10.7586/jkbns.25.077

**Published:** 2026-02-23

**Authors:** Hyun Bin Kim, Eun Ji Seo

**Affiliations:** 1 Department of Emergency Nursing, Gangnam Severance Hospital, Seoul, Korea; 1 강남세브란스병원 응급진료파트; 2 College of Nursing · Research Institute of Nursing Science, Ajou University, Suwon, Korea; 2 아주대학교 간호대학 · 간호과학연구소

**Keywords:** Neoplasms, Emergency medical services, Length of stay, 암, 응급의료서비스, 체류시간

## Abstract

**Purpose:**

This study aimed to characterize cancer patients presenting to the emergency department (ED) and to identify factors associated with ED length of stay (LOS) and 72-hour revisits.

**Methods:**

This retrospective study analyzed the medical records of adult cancer patients who visited the ED of a tertiary hospital in Seoul between June and November 2023. Among 21,495 ED visits, 2,225 met the inclusion criteria. Variables included general characteristics, cancer-related characteristics, and ED visit–related characteristics. Outcomes were ED LOS and 72-hour revisits. Data were analyzed using the t-test, analysis of variance, chi-square test, multiple linear regression, and logistic regression.

**Results:**

The mean age was 64.75 ± 14.85 years, and 51.0% of patients were men. Gastrointestinal cancers were the most common cancer type (49.6%), and abdominal pain was the most frequent chief complaint (17.4%). The mean ED LOS was 245.93 ± 189.79 minutes, and the 72-hour revisit rate was 5.4%. Factors associated with longer ED LOS included older age, previous ED visits, off-hours arrival, higher acuity, ambulance transport, abnormal respiratory rate, and administration of nonsteroidal anti-inflammatory drugs, acetaminophen, or opioids. Factors influencing 72-hour revisits included the absence of a do-not-resuscitate (DNR) order, abnormal systolic blood pressure, and admission.

**Conclusion:**

For older cancer patients with high acuity who visit the ED by ambulance during off-hours, early assessment of dyspnea and prior ED utilization, along with prompt symptom-based pharmacologic interventions, is recommended. At discharge, providing education tailored to the patient’s clinical condition, including DNR status, admission status, and vital signs, and ensuring early outpatient or telephone follow-up may strengthen continuity of care and reduce short-term ED revisits.

## 서론

### 1. 연구의 필요성

암은 우리나라 사망원인 1위(10만명당 162.7명)로서, 2위와 3위인 뇌혈관질환, 심장질환의 수를 합친 것보다 많다[1]. 최근 수년간 암의 유병률 역시 꾸준히 증가하여 전체 암 환자 규모가 지속적으로 확대되고 있다[2]. 또한 2021년 우리나라 암 5년 상대생존율은 72.1%로 20년 동안 약 2배 정도 향상되었는데[3], 암 환자의 치료 후 생존 기간 연장에 따라 지속적인 관리가 필요한 환자 수가 증가하고 있음을 의미한다. 이와 같이 암을 진단받은 환자가 단순히 증가하는 것을 넘어 장기 생존군의 확대로 의료체계 내에서 환자 분포가 지속적으로 커지고 있다.

암의 치료가 고도화, 다변화되면서[4] 치료 관련 독성의 스펙트럼도 변화하고 있다. 방사선 치료 후 폐렴, 화학요법 후 심혈관계 독성, 말초신경병증, 인지기능장애 등의 급성 평가가 필요한 상황이 다양하게 발생하고 있다[5,6]. 그로 인한 여러 증상으로 암 환자는 응급실에 방문하게 되며, 통증, 발열, 오심, 구토, 호흡곤란 등이 흔한 주호소이다[7]. 우리나라 암 환자의 응급실 내원 비율 또한 1997년 17.4%에서 2009년 25.5%로 증가하였는데[8,9], 이는 미국의 2012년 4.2%보다 높은 수준이다[7,10]. 더불어 응급실 다방문 환자군에서도 암 환자의 방문 빈도는 다른 만성 질환군에 비해 높게 나타났다[11].

암 환자의 경우 응급실 총 체류시간도 다른 만성질환 환자보다 유의하게 더 길었다[8,11]. 암으로 치료받았던 환자가 응급실에 내원하면, 암이라는 기저질환에 의한 타과 협의진료 필수성과 증상에 대한 복잡한 병태생리로 인해 의사결정이 지연될 수 있어 체류시간이 증가할 수 있다[12,13]. 내원 당시 증상의 급성기가 해결되더라도 퇴원 후 관리 계획이나 다른 의료기관으로의 전원 필요성 고려 등 응급실 퇴원 준비 과정이 길어질 수 있다[13]. 더불어, 응급실과 같은 긴박하고 압박된 상황에서 의료진은 주호소의 급성기만 지나면 퇴원을 계획하게 되는 경우도 있는데, 시공간의 제약으로 인해 응급실 퇴원 시 제공되는 자가관리 지침을 환자가 충분히 이해하지 못하는 등 퇴원교육에 대한 고려도 충분히 이루어지지 않고 있다[14,15]. 이러한 맥락에서 암 환자의 체류시간과 재방문은 다른 환자에 비해 많거나 길어지는 경향을 보일 수 있다. 특히 응급실 재방문 중에서도 72시간 이내 재방문은 단기 재방문으로, 응급의료서비스의 질 향상, 업무 효율성, 환자 안전을 평가하기 위한 대표적 지표로 활용되어 왔다[16].

이에 본 연구는 응급실에 방문하는 암 환자의 특성을 파악하고 응급실 체류시간 및 72시간 이내 응급실 재방문과 관련 있는 특성을 확인하여, 응급실 체류시간 연장 및 단기 재방문 위험이 높은 고위험군을 선별하기 위한 기초자료를 마련하고자 한다.

### 2. 연구의 목적

본 연구의 목적은 응급실에 내원하는 암 환자의 특성을 파악하여 응급실 체류시간과 응급실 재방문 관련요인을 확인하는 것이다. 구체적인 목표는 다음과 같다.

첫째, 암 환자의 일반적 특성과 암 관련 특성, 응급실 내원 관련 특성, 응급실 체류시간, 72시간 이내 응급실 재방문 여부에 대해 파악한다.

둘째, 암 환자의 응급실 체류시간과 응급실 재방문에 영향을 미치는 요인을 파악한다.

## 연구 방법

### 1. 연구 설계

본 연구는 서울시 소재 일 상급종합병원 응급실에 내원한 환자 중 암을 기저질환으로 가진 환자를 대상으로, 응급실 체류시간과 응급실 재방문 영향요인을 규명하기 위한 후향적 서술적 조사연구이다.

### 2. 연구 대상

본 연구의 대상자는 응급실에 내원한 성인 암 환자이다. 혈액암은 질병 경로 및 치료 요구가 고형암과 상이하므로[17] 연구 대상에서 제외하였다. 구체적인 대상자 선정기준은 다음과 같다.

1) 18세 이상의 성인

2) 암을 기저질환으로 가진 환자

연구대상자 제외기준은 다음과 같다.

1) 도착시 이미 사망한 경우

2) 임산부

3) 혈액암 환자

4) 진료결과 전원 또는 사망

5) 기록이 미비한 경우

2023년 6월부터 11월까지 일 상급종합병원 응급실에 내원한 환자 21,495명 중 암을 기저질환으로 가진 대상자는 2,546명이었다. 이들 중 제외 기준인 18세 미만(n = 7), 도착 시 사망(n = 5), 임산부(n = 0), 혈액암(n = 109), 진료결과 전원 또는 사망(n = 26), 기록 미비(n = 174)인 대상자를 제외하여 최종 2,225명을 대상으로 연구를 수행하였다.

### 3. 연구 도구

#### 1) 일반적 특성

대상자의 일반적 특성은 선행연구[18-20]를 참고하여, 성별, 연령, 거주지역, 이전 응급실 내원 여부, 내원 전 6개월 이내 입원력, 동반질환(고혈압, 당뇨)의 7항목으로 수집하였다.

#### 2) 암 관련 특성

대상자의 암 관련 특성은 선행연구[18-20]를 참고하여, 진단받은 암, 전이 여부, DNR (do-not-resuscitate) 여부, 항암 치료 여부, 방사선 치료 여부, 수술 여부의 6항목을 수집하였다. 진단받은 암은 전자의무기록에서 추출가능한 진단명을 기준으로 신체계통(소화기계, 비뇨생식기계/부인과, 두경부, 유방, 폐)으로 구분하였다. 암 진단명에 전이가 명시된 경우는 전이 여부 변수로 반영하였고, 전이가 명시되지 않은 진단명은 원발암으로 간주하여 해당 장기 범주에 포함하였다.

#### 3) 응급실 내원 관련 특성

대상자의 응급실 내원 관련 특성은 선행연구[18-20]를 참고하여, 내원시간, 중증도(Korean Triage and Acuity Scale, KTAS), 내원수단, 주호소, 내원 당시 활력징후, 산소포화도, 내원 후 약물중재(non-steroidal anti-inflammatory drugs [NSAIDs], acetaminophen, opioid, 항구토제) 여부, 산소중재 여부, 진료결과(입원, 퇴원)를 수집하였다. 활력징후 표준화 기초조사 연구[21]에 따라, 내원 당시 활력징후를 정상 범위 여부로 구분하였다. 정상범위는 맥박 60-100회/분, 체온 36.1~37.2°C, 수축기혈압 90~119 mmHg, 산소포화도 95%~100%, 호흡 수 12-20회/분이었다.

#### 4) 결과 변수

본 연구에서 수집한 대상자의 결과 변수는 응급실 체류시간과 72시간 이내 응급실 재방문 유무이다. 체류시간은 응급실 접수 이후 입원(입원 결정 시점) 또는 퇴원까지의 시간을 조사하였다. 응급실 재방문은 퇴원시간 이후부터 72시간 이내 해당 기관 응급실에 재방문 한 경우를 의미한다.

### 4. 자료 수집

본 연구는 서울시 소재 강남세브란스병원에서 운영하는 Analysis Portal 시스템을 통해 자료를 수집하였다. 본 시스템은 진단, 수술, 처방, 처치, 검사, 임상관찰, 전자의무기록 등의 빅데이터를 원하는 조건으로 추출 가능한 시스템이다. 기관생명윤리위원회(institutional review board, IRB) 승인 후 해당 Portal 접근과 자료 추출에 대한 승인을 받아 2025년 6월 1일부터 2025년 6월 30일의 기간 동안 연구자가 직접 추출하였다. 코로나19 팬데믹과 국내 의료계 상황으로 인해 연구대상자는 2023년 6월부터 11월까지 응급실에 내원한 환자 중 본 연구대상자 선정기준에 부합한 대상자의 자료를 추출하였다. 추출한 정보에는 자동적으로 환자 이름은 제외되며 환자 등록번호 역시 개인 식별 자료가 아닌 임의 부여된 연구용 등록번호로 제공되었다.

### 5. 자료 분석

수집된 자료는 SPSS version 26.0 (IBM Corp., Armonk, NY, USA) 프로그램을 이용하여 다음과 같이 분석하였다.

1) 대상자의 일반적 특성, 암 관련 특성, 응급실 내원 관련 특성은 빈도와 백분율, 평균과 표준 편차 등의 기술통계로 분석하였다.

2) 대상자의 일반적 특성, 암 관련 특성, 응급실 내원 관련 특성에 따라 응급실 체류시간과 응급실 재방문 유무의 차이는 independent t-test, one-way analysis of variance, 교차분석을 이용하였고, 사후 검정으로 Scheffe's test를 실시하였다.

3) 체류시간의 영향요인을 분석하기 위해 다중회귀분석을 시행하였다. 체류시간의 분포가 오른쪽으로 치우쳐진 형태이므로, 정규성 확보를 위해 log 변환[22]을 적용한 후 분석을 실시하였다.

4) 응급실 재방문의 영향요인을 분석하기 위해 로지스틱 회귀분석을 시행하였다.

### 6. 윤리적 고려

본 연구는 강남세브란스병원의 IRB의 승인을 받았다(IRB No. 3-2024-0469). 연구 대상자의 개인정보는 자료추출 과정에서 익명화되었으며, 자료는 연구 목적 이외에는 활용하지 않았다. 본 연구는 후향적 의무기록 연구로서 대상자 동의는 면제되었다.

## 연구 결과

### 1. 응급실 내원 암 환자의 특성

대상자의 일반적 특성은 남자가 51.0%이었으며, 60~69세인 대상자가 가장 많았다. 이전 응급실 내원 경험이 있는 환자는 70.7%, 6개월 이내 입원력이 있는 환자는 58.9%였다.

대상자의 암 관련 특성으로, 소화기계 암이 49.6%으로 절반가량 차지하였다. 전체의 53.8%가 항암 치료를 받았고, 67.6%는 수술 치료를 받았다. DNR을 작성한 대상자는 28.4%이었다.

대상자의 응급실 내원 관련 특성으로, 60.8%가 일과시간(9~18시)에 내원하였고 중증도가 높은 대상자(KTAS 1~3단계)가 69.3%였다. 응급실 내원 시 주호소는 복통(17.4%)이 가장 많았고, 다음으로는 발열 13.5%, 호흡곤란 7.7% 순이었다. 활력징후의 경우 대부분 정상 범위였으나, 수축기혈압은 고혈압이나 저혈압 범주인 환자가 75.3%로 더 많았다. 내원 후 진행한 약물 중재는 대부분 진통/해열제(NSAIDs, acetaminophen, opioid)와 항구토제였고, 이 중 acetaminophen 투여(20.7%)가 가장 많았다. 암 환자의 55.9%가 응급실 진료 후 입원하였다(Table 1).

### 2. 응급실 내원 암 환자의 특성에 따른 체류시간 및 72시간 이내 응급실 재방문 차이

대상자의 응급실 체류시간은 평균 245.93 ± 189.79분(log 변환 시, 5.23 ± 0.81)이었다. 72시간 응급실 재방문은 5.4%이었다(Table 2). 응급실 내원 암 환자의 특성에 따른 응급실 체류시간의 차이(Supplementary Table 1)와 72시간 이내 응급실 재방문의 차이(Supplementary Table 2)는 다음과 같다.

대상자의 일반적 특성에 따른 응급실 체류시간의 차이는 연령, 거주지역, 이전 응급실 내원 여부, 고혈압, 당뇨 여부에서 유의하였다. 암 관련 특성에서는 소화기계 암, 유방암, 또는 폐암인 경우, DNR 여부, 항암 치료 여부에 따라 응급실 체류시간에 유의한 차이를 보였다. 응급실 내원 관련 특성에 따른 응급실 체류시간의 차이는 내원시간, 중증도, 내원수단, 주호소 중 abdominal pain, fever, dyspnea, 활력징후 중 맥박, 체온, 호흡수, 산소포화도, 응급실에서의 약물 중재, 산소 중재, 진료결과에서 유의하였다(Supplementary Table 1).

72시간 이내 응급실 재방문은 일반적 특성에서 6개월 이내 입원력, 암 관련 특성에서 DNR 여부가 유의한 차이를 보였다. 응급실 내원 관련 특성에서는 내원시간, 수축기혈압, 진료결과에 따라 유의한 차이를 보였다(Supplementary Table 2).

### 3. 응급실 체류시간에 대한 다중회귀분석

응급실 체류시간 영향요인을 파악하기 위해 단변량 분석에서 유의한 차이를 보인 특성(연령, 거주지역, 이전 응급실 내원 여부, 고혈압, 당뇨, 소화기계 암, 유방암, 폐암, DNR 여부, 항암 여부, 내원시간, 중증도, 내원수단, abdominal pain, fever, dyspnea, 맥박, 체온, 산소포화도, 호흡수, NSAIDs, acetaminophen, opioid, 항구토제, 산소 중재, 진료결과)을 포함하여 다중회귀분석을 시행하였다. 회귀분석의 기본 가정 충족을 점검하기 위해 선형성, 정규성, 다중공선성, 자기 상관성 검증을 실시하였다. 독립변수 간 상관분석 결과, 모든 변수의 상관계수는 0.8 미만으로 나타나 분석에 포함하였다. Durbin-Watson 통계량은 1.92로 2에 근사한 값을 보여 잔차의 자기상관은 없었다. 공차 한계는 .58~.95로 .10 이상이었고, variance inflation factors는 1.05~1.73으로 10 미만으로 다중공선성 문제도 없어, 회귀분석을 위한 가정을 충족하였다.

회귀모형은 통계적으로 유의하였으며(F = 13.17, *p* < .001), 설명력은 13%로 나타났다(*R*^2^ = .14, *adj.* *R*^2^ = .13). 응급실 체류시간에 유의한 영향을 미친 변수는 acetaminophen (β = .18), 중증도(β = .16), 내원수단(β = .12), opioid (β = .11), 호흡수(β = .07), 연령(β = .05), 내원시간(β = .05), 이전 응급실 내원 여부(β = .05), NSAIDs (β = .04)였다. 즉, 연령이 증가할수록, 이전에 응급실에 내원한 경험이 있는 경우, 일과시간 외에 내원하는 경우, 중증도가 응급인 경우, 구급차를 이용하여 내원한 경우에 응급실 체류시간이 증가하였다. 활력징후 중 호흡수가 정상 범주가 아닌 경우에 체류시간이 더 길었고, 응급실에서 NSAIDs, acetaminophen, 또는 opioid를 투여받은 경우에 체류시간이 길었다(Table 3).

### 4. 72시간 이내 응급실 재방문에 대한 로지스틱 회귀분석

응급실 재방문 영향요인을 파악하기 위해 단변량 분석에서 유의한 차이를 보인 특성(6개월 이내 입원력, DNR 여부, 내원시간, 수축기혈압, 진료결과)을 투입하여 로지스틱 회귀분석을 시행하였다. 본 회귀분석은 통계적으로 유의하였고(*χ*^2^ = 49.24, *p* < .001), 모형은 자료에 잘 부합하였으며(Hosmer & Lemeshow *χ*^2^ = 2.80, *p* = .946), 설명력은 6% (Nagelkerke *R*^2^ = .06)이었다.

로지스틱 회귀분석 결과, 퇴원 환자에 비해 입원 환자의 재방문 가능성이 2.77배 높았으며(odds ration [OR] = 2.77), 수축기혈압이 정상인 환자에 비해 비정상에 속한 환자의 재방문 가능성이 1.50배 높았다(OR = 1.50). 또한 DNR을 받지 않은 환자는 받은 환자에 비해 재방문 가능성이 높았다(OR = 0.37)(Table 4).

## 논의

본 연구는 응급실에 내원하는 암 환자의 특성을 파악하고, 응급실 체류시간 및 재방문과 관련된 요인을 확인하여 고위험군을 선별하기 위한 기초자료를 마련하고자 시행되었다.

응급실에 내원하는 암 환자의 체류시간은 평균 245.93 ± 189.79분(4.10 ± 3.16시간)이었으며, 이는 국내 단일 암 전문병원 응급실을 대상으로 한 연구[23]의 5.8시간보다 짧았고, 미국의 전국 단위 National Hospital Ambulatory Medical Care Survey 자료를 이용한 연구[24]의 4.9시간과 비교해서도 짧았다. 이러한 차이는 각 연구에서 응급실 체류시간을 정의한 방식의 차이에서 비롯된 것으로 보인다. 선행 연구[23,24]에서는 체류시간을 응급실에 도착한 시점부터 실제 응급실 퇴실(퇴원 또는 병동으로 이동)까지로 정의하였다. 그러나 단일 암 전문병원 연구[23]는 입원 병상 부족 등 구조적 요인이 체류시간 연장과 관련될 수 있음을 지적했다. 본 연구는 이러한 외부 요인 영향을 배제하고자, 응급실 도착부터 입원결정 시점까지를 응급실 체류시간으로 정의하였다. 또한 72시간 이내 응급실 재방문 비율은 5.4%이었다. 항암화학요법을 받는 암 환자만을 대상으로 한 국내 선행연구[25]의 8.2%보다 낮았다. 본 연구결과는 최근 치료 환경의 개선과 예방적 지지요법의 표준화 등으로 인해, 치료에 따른 부작용이 감소하는 경향으로 해석된다[26-28].

응급실에 내원하는 암 환자는 소화기계 암 환자가 가장 많았다. 이는 전국 응급실 자료를 활용한 연구[18,29]와 일관된 결과이다. 2022년 국가 암 등록통계[30]에서도 위암과 대장암은 상위 발생 암으로 가장 많은 비중을 차지하였다. 응급실에 내원하는 암 환자들의 주호소는 복통, 발열 순이었는데, 선행연구[31]에서도 통증이 암 환자가 응급실에 방문하는 가장 흔한 증상이었다. 암 환자의 통증은 질병 자체뿐 아니라 다양한 치료 과정에서도 발생할 수 있으며, 일부는 만성화되기도 한다[32]. 이러한 통증은 일상 기능과 삶의 질을 저하시키므로[32], 암 치료 전후 단계에서의 적절한 통증 관리가 필수적이다.

내원 직후 활력징후는 대체로 정상 범위였으나, 수축기혈압은 비정상인 경우가 75.3%로 더 많았다. 응급실 전체 환자를 대상으로 한 연구[20]에서도 수축기혈압의 비정상 비율이 상대적으로 높게 보고되었다. 고혈압의 경우, 응급실 도착 시 대상자 증상과 불안에 따른 교감신경 항진으로 일시적 수축기혈압 상승이 관찰될 수 있다[33]. 반면 저혈압은 암 환자에서 패혈증, 호중구감소성 발열 등과 밀접하게 관련되며, 특히 호중구감소 상태의 중증 감염에서는 저혈압이 초기 징후로 관찰될 수 있다[34]. 이와 같이, 암 환자에서 수축기혈압의 비정상은 내원 시 상태의 위중함을 시사할 수 있어, 초기 평가에서 다른 활력징후보다 혈압 변화에 대한 면밀한 관찰이 요구된다.

응급실 체류시간에 영향을 미치는 요인은 연령, 이전 응급실 내원 여부, 내원시간, 중증도, 내원수단, 호흡수, NSAIDs, acetaminophen, opioid 투여였다. 연령은 증가할수록 응급실 체류시간이 더 증가하였다. 고령 환자는 기저질환이 다수 존재하고 비특이적 증상으로 내원하는 경우가 많아 진단과 처치 등 의사결정 과정에 더 많은 시간이 필요한 임상적 복잡군으로 볼 수 있다[35]. 과거 응급실을 이용한 경험이 있는 환자는 그렇지 않은 환자보다 체류시간이 길었는데, 이는 증상의 재발이나 만성화 등으로 인해 임상적 복잡성이 좀 더 높을 수 있기 때문으로 해석된다[36]. 즉, 이전 응급실 내원 경험이 있다는 것은 재발 위험 및 관리 부담이 높은 환자군을 조기에 식별할 수 있는 중요한 임상적 표지자로 활용될 수 있다. 일과시간 외 내원인 경우 체류시간이 유의하게 증가하였다. 영국 응급실 자료를 이용한 코호트 연구[37] 또한 일과시간 외 시간대에 전문의 협진 및 검사 수행이 지연되는 등 운영상 제약이 존재함을 보고하였다.

본 연구에서 중증도가 높을수록 암 환자의 응급실 체류시간은 유의하게 길었다. 이는 표준화된 신속 치료 경로(예: 심근경색의 door-to-balloon, 뇌경색의 door-to-needle)와 패스트트랙이 정착된 질환에서 상대적으로 체류시간이 짧게 관찰되는 결과[20]와 대조적이다. 한편, 구급차로 내원한 환자는 스스로 혹은 타인에 의해 긴급 대응이 필요하다고 인식된 집단으로, 비정상 호흡수를 보인 환자와 함께 임상적 긴박성과 생리적 불안정 가능성이 높은 군으로 해석된다. 특히 활력징후 중 호흡수 이상은 환자 상태 악화의 조기 신호로서 응급실 초기 평가와 모니터링 시 특히 고려할 필요가 있다. 이러한 특성을 보인 환자의 체류시간 연장은 단순한 프로세스 지연이 아니라, 중증도에 따른 자원 집중과정의 결과로 이해할 수 있다. 따라서 암 환자에서 중증도와 내원 특성에 기반한 표준화된 평가 및 처치 경로 구축은 환자 관리 효율성을 높이는 핵심 전략이 될 수 있다.

약물 중재 측면에서 NSAIDs, acetaminophen, opioid를 투여받은 환자는 비투여군보다 응급실 체류시간이 길었고, 그 중 acetaminophen 투여군에서 가장 체류시간이 길었다. Acetaminophen은 임상에서 해열제로 널리 사용되는데, 암 환자의 발열은 패혈증 여부 등 감염 평가와 항생제 사용이 동반되는 경우가 많아 추가 체류시간을 요구할 수 있다[38]. Opioid는 중증도 이상의 통증에서 주로 사용되어 투여 전 금기 확인과 용량 결정, 투여 후 활력징후 모니터링 등의 추가 중재가 필요하기 때문으로 해석된다. 한편, 진통제 투여까지의 시간이 짧을수록 전체 체류시간이 단축된다는 연구[39,40]는 초기 통증부터 약물 투여, 신속한 재평가가 체류시간 관리에 중요한 부분임을 시사한다. 따라서 암 환자의 주요 주호소로 드러나는 통증 및 발열을 관리하는 프로세스 구축이 체류시간 단축을 위한 핵심 전략으로 제시된다.

72시간 이내 응급실 재방문에 영향을 미치는 요인은 DNR 여부와 응급실 내원 시 비정상 수축기혈압, 응급실 진료결과가 입원인 경우였다. DNR을 작성하지 않은 환자에서 재방문이 높았다는 결과는 적극적 치료를 선호하거나 응급의료 이용 성향이 다른 집단일 가능성을 시사한다. 반면 DNR을 작성한 환자는 유사한 급성 증상이 발생해도 완화의료나 장기요양 등 대체 치료 및 돌봄 경로를 선택할 가능성이 커 응급실 방문을 하지 않을 수 있다[41]. 내원 당시 비정상 수축기협압은 72시간 이내 응급실 재방문을 증가시켰다. 대만 단일 3차병원 성인 응급실 환자를 대상으로 한 연구[42]에서도 비정상 수축기혈압의 환자들은 중환자실 입원이나 사망에 이른 고위험 응급실 재방문군으로 보고되었다. 즉, 응급실 내원 당시 수축기혈압의 이상은 응급실 내 중증도 판단뿐 아니라, 퇴원 후 단기 악화 가능성을 조기에 예측하는 지표로 활용될 수 있다. 진료결과에서는 입원한 경우가 퇴원보다 72시간 이내 응급실 재방문 가능성이 높았다. 입원 결정 자체가 동반질환과 높은 중증도 등 임상적 복합성을 반영한 결과임을 시사한다[43,44]. 선행연구에 따르면 72시간 재방문은 기존 증상의 지속 또는 재악화로 설명되며[45], 퇴원 지시 이해의 부족 또한 단기 재방문에 기여할 수 있다고 한다[15,46]. 그러므로, 암 환자의 응급실 내원 시 활력징후(특히 수축기혈압) 감시와 치료 목표(DNR 여부)확인이 중요하고, 퇴원 시점에는 주호소의 충분한 안정화 확인과 악화 시 즉각 재내원 지침 제공, 퇴원 후 48~72시간 이내 외래 또는 전화 추적을 포함한 표준화된 퇴원교육 체계가 필요하다. 이러한 접근은 응급실에서도 집중적 퇴원관리 프로그램이 응급실 재방문을 줄이는 핵심 전략이 될 수 있음을 시사한다[15,46].

본 연구에서 응급실 체류시간과 72시간 이내 응급실 재방문에 대한 영향요인이 도출되었으나, 이들의 설명력은 각각 13%와 6%로 비교적 낮게 나타났다. 암 환자의 일반적, 암 관련, 응급실 내원 관련 특성을 폭넓게 검토하였음에도 불구하고, 분석이 주로 대상자 요인에 집중되었기 때문으로 해석된다. 특히 응급실 체류시간은 환자 요인 뿐 아니라 인력 배치, 검사와 협진 체계, 병상 가용성 같은 시스템 요인을 함께 고려하는 것이 필요한 지표이다[37]. 다만, 본 연구에서 제시된 암 환자의 중증도, 내원 특성, 주요 주호소에 기반한 표준화된 평가 및 처치 경로가 구축된다면 향후 응급실 시스템 요인 전반에도 변화가 나타날 수 있을 것이다.

본 연구는 여러 가지 제한점이 있다. 첫째, 일개 상급종합병원의 일부 기간 자료에 근거한 후향적 연구이기 때문에, 결과를 일반화하기에는 제한이 있다. 또한 연구 기간 동안 환자가 다른 응급실을 방문했을 가능성에 대한 자료가 포함되지 않아 불완전할 수 있다. 둘째, 본 연구의 자료는 연구 대상 기관의 Analysis Portal 시스템에 입력된 정보로 한정되었다. 고혈압과 당뇨와 같은 주요 동반질환은 구조화된 항목으로 추출이 가능하였으나, 그 외 동반질환은 대상자의 표현에 따라 통일되지 않은 용어로 다양하게 기재되어 있어 추출 및 분류의 신뢰성을 확보할 수 없었다. 이로 인해 다양한 동반질환을 고려하지 못하였다. 셋째, 암 환자의 원발암 장기, 전이 장기, 전이 시점의 병기와 같이 응급실 내원의 주호소와 관련될 수 있는 요소들을 고려할 필요가 있으나, 본 연구에서 활용한 자료 추출 시스템에서는 이러한 세부 정보를 확인할 수 없었다. 이에 따라 응급실 이용 패턴의 복잡성을 충분히 반영하지 못하였을 수 있다. 넷째, 본 연구는 암 환자만을 대상으로 한 코호트 분석으로, 동기간 비암 환자군과의 직접 비교 분석은 본 연구의 범위를 넘어 수행하지 않았다. 이로 인해 응급실 체류시간의 연장이나 72시간 이내 재방문 증가가 암 환자 특이 요인에 기안한 것인지, 응급실 전반의 혼잡 및 운영 요인 등 비특이적 요인의 영향인지를 분리하여 해석하는데 한계가 있다. 다섯째, 응급실 체류시간과 재방문에 대한 두 모형 모두 유의한 변수들이 도출되었으나 설명력이 낮았다. 향후 연구에서는 대상자 요인 뿐 아니라 이들 결과에 영향을 미칠 수 있는 응급실 환경 요인 등 다양한 변수를 고려할 필요가 있다.

## 결론

본 연구는 응급실에 내원하는 암 환자의 특성과 임상 결과(응급실 체류시간, 72시간 이내 재방문)의 영향요인을 규명하고자 하였다. 응급실 체류시간은 연령, 이전 응급실 내원 여부, 내원시간, 중증도, 내원수단, 호흡수, 내원 후 NSAIDs, acetaminophen, opioid 투여와 유의한 관련이 있었다. 72시간 이내 응급실 재방문은 진료 후 입원, 수축기혈압, DNR 여부와 관련이 있었다. 이러한 결과는 KTAS 1~3단계의 고령 암 환자가 일과 외 시간에 구급차로 내원하면, 호흡곤란 여부와 이전 응급실 내원 여부를 우선적으로 확인하고 주호소에 따른 약물중재를 신속히 시행할 필요성을 시사한다. 이를 위해, 중증도와 내원 특성(호흡곤란, 이전 응급실 내원 여부 등)에 따른 증상 관리의 표준화 프로세스를 구축하고, 통증 및 발열 중심의 신속한 대응 체계를 마련할 필요가 있다. 또한, 응급실 퇴원 시에는 대상자의 상태(DNR 및 입원 여부) 및 내원 시 활력징후에 따른 적절한 퇴원교육과 조기 외래 추적 관리가 강조되어야 하며, 이는 돌봄 연속성을 강화하여 응급실 재방문을 줄이는 전략이 될 수 있을 것이다.

## Figures and Tables

**Table 1.** Clinical Characteristics of Cancer Patients Presenting to the Emergency Department (N = 2,225)

Characteristics	Categories	n (%)
General characteristics		
Sex	Men	1,135 (51.0)
	Women	1,090 (49.0)
Age (years)	≤ 49	378 (17.0)
	50 ∼ 59	359 (16.1)
	60 ∼ 69	605 (27.2)
	70 ∼ 79	499 (22.4)
	≥ 80	384 (17.3)
Residence	Metropolitan	1,753 (78.8)
	Non-metropolitan	472 (21.2)
Prior ED visit	First visit	652 (29.3)
	≥ 1 prior visits	1,573 (70.7)
Prior hospitalization (within 6 months) (yes)		1,311 (58.9)
Comorbidities		
Hypertension (yes)		672 (30.2)
Diabetic mellitus (yes)		426 (19.1)
Cancer-related characteristics		
Cancer diagnosis		
Gastrointestinal (yes)		1,103 (49.6)
Genitourinary/gynecologic (yes)		387 (17.4)
Head and neck (yes)		370 (16.6)
Breast (yes)		282 (12.7)
Lung (yes)		172 (7.7)
Metastasis (yes)		690 (31.0)
DNR status (yes)		631 (28.4)
Chemotherapy (yes)		1,196 (53.8)
Radiotherapy (yes)		817 (36.7)
Surgery (yes)		1,505 (67.6)
ED visit-related characteristics		
Arrival time	Business hours (09:00~18:00)	1,353 (60.8)
	Off-hours	872 (39.2)
Acuity	Emergent (KTAS 1,2,3)	1,541 (69.3)
	Non-emergent (KTAS 4,5)	684 (30.7)
Mode of arrival	Walk-in	1,791 (80.5)
	Ambulance (119, 129)	434 (19.5)
Chief complaint		
Abdominal pain (yes)		388 (17.4)
Fever (yes)		301 (13.5)
Dyspnea (yes)		171 (7.7)
General weakness (yes)		131 (5.9)
Nausea and vomiting (yes)		66 (3.0)
Pulse rate (/min)	Normal (60 ∼ 100)	1,529 (68.7)
	Abnormal range (< 60 and > 100)	696 (31.3)
Body temperature (℃)	Normal (36.1 ∼ 37.2)	1,515 (68.1)
	Abnormal range (< 36.1 and >37.2)	710 (31.9)
Systolic blood pressure (mmHg)	Normal (90 ∼ 119)	549 (24.7)
	Abnormal range (< 90 and ≥ 120)	1,676 (75.3)
Oxygen saturation (%)	Normal (95 ∼ 100)	2,112 (94.9)
	Abnormal range (< 95)	113 (5.1)
Respiratory rate (/min)	Normal (12 ∼ 20)	2,163 (97.2)
	Abnormal range (< 12 and > 20)	62 (2.8)
NSAIDs (yes)		113 (5.1)
Acetaminophen (yes)		461 (20.7)
Opioid (yes)		298 (13.4)
Antiemetics (yes)		248 (11.1)
Oxygen therapy in the ED (yes)		125 (5.6)
ED disposition	Admission	1,243 (55.9)
	Discharge	982 (44.1)

ED = Emergency department; DNR = Do-not-resuscitate; KTAS = Korean Triage and Acuity Scale; NSAIDs = Non-steroidal anti-inflammatory drug.

**Table 2.** Emergency Department Length of Stay and Revisits within 72 Hours (N = 2,225)

Clinical outcomes	Categories	n (%)	M ± SD (range)
ED length of stay (minutes)			245.93 ± 189.79 (12.00 ∼ 1998.00)
Log-transformed			5.23 ± 0.81 (2.48 ∼ 7.60)
ED revisit within 72 hours	Yes	121 (5.4)	
	No	2,104 (94.6)	

ED = Emergency department; M = Mean; SD = Standard deviation.

**Table 3.** Factors Influencing Emergency Department Length of Stay (N = 2,225)

Variables	B	SE	β	t	*p*
(Constant)	4.55	0.09		50.63	< .001
Age	0.00	0.00	.05	2.38	.017
Residence (non-metropolitan)	-0.06	0.04	-.03	-1.38	.168
Prior emergency department visit (≥1 prior visit)	0.08	0.04	.05	2.23	.026
Hypertension (yes)	0.01	0.04	.01	0.24	.808
Diabetic mellitus (yes)	0.06	0.04	.03	1.44	.150
Gastrointestinal cancer (yes)	0.02	0.04	.01	0.40	.690
Breast cancer (yes)	-0.05	0.06	-.02	-0.98	.328
Lung cancer (yes)	0.04	0.07	.01	0.56	.577
DNR status (yes)	0.03	0.04	.01	0.64	.521
Chemotherapy (yes)	-0.00	0.04	-.00	-0.02	.982
Arrival time (off-hours)	0.08	0.03	.05	2.24	.025
Acuity (emergent)	0.29	0.04	.16	7.58	<.001
Mode of arrival (ambulance)	0.25	0.04	.12	5.81	<.001
Abdominal pain (yes)	0.04	0.05	.02	0.77	.443
Fever (yes)	0.01	0.06	.00	0.10	.920
Dyspnea (yes)	-0.01	0.07	-.00	-0.15	.881
Pulse rate (/min) (< 60 and > 100)	0.05	0.04	.03	1.27	.203
Body temperature (℃) (< 36.1 and > 37.2)	-0.05	0.04	-.03	-1.25	.212
Oxygen saturation (%) (< 95)	-0.02	0.08	-.01	-0.22	.830
Respiratory rate (/min) (< 12 and > 20)	0.32	0.11	.07	3.02	.003
NSAIDs (yes)	0.15	0.08	.04	2.03	.043
Acetaminophen (yes)	0.36	0.05	.18	6.88	< .001
Opioid (yes)	0.27	0.05	.11	5.28	< .001
Antiemetics (yes)	0.08	0.05	.03	1.43	.152
Oxygen therapy in the emergency department (yes)	0.03	0.08	.01	0.43	.665
Emergency department disposition (admission)	-0.02	0.04	-.01	-0.43	.671
R²(Adj R²)		.14 (.13)			
F(*p)*		13.17 (<.001)			

SE = Standard error; DNR = Do-not-resuscitate; NSAIDs = Non-steroidal anti-inflammatory drug. Reference = residence (metropolitan); prior emergency department visit (first visit); hypertension (no); diabetes mellitus (no); gastrointestinal cancer (no); breast cancer (no); lung cancer (no); DNR status (no); chemotherapy (no); arrival time (business hours); acuity (non-emergent); mode of arrival (walk-in); abdominal pain (no); fever (no); dyspnea (no); pulse rate (normal [60 ~ 100]); body temperature (normal [36.1 ∼ 37.2]); oxygen saturation (normal [95 ~ 100]); respiratory rate (normal [12 ~ 20]); NSAIDs (no); acetaminophen (no); opioid (no); antiemetics (no); oxygen therapy in the emergency department (no); emergency department disposition (discharge).

**Table 4.** Factors Influencing Emergency Department Revisits within 72 Hours (N = 2,225)

Variables	OR	95% CI	*p*
Prior hospitalization (within 6 months) (yes)	0.71	0.47 ~ 1.08	.106
DNR status (yes)	0.37	0.24 ~ 0.56	<.001
Arrival time (off-hours)	0.70	0.48 ~ 1.02	.060
Systolic blood pressure (mmHg) (Normal [90 ~ 119])	1.50	1.01 ~ 2.24	.045
Emergency department disposition (admission)	2.77	1.83 ~ 4.17	<.001
χ² = 49.24, *p *< .001, Nagelkerke R² = .06			
Hosmer & Lemeshow test: χ² = 2.80 (*p* = .946)			

OR = Odds ratio; CI = Confidence interval; DNR = Do-not-resuscitate. Reference = prior hospitalization (within 6 months) (no); DNR status (no); arrival time (business hours); systolic blood pressure (abnormal range [< 90 and ≥ 120]); emergency department disposition (discharge).

## Data Availability

Please contact the corresponding author for data availability.

## Supplementary Materials

Supplementary Table 1. Differences in Emergency Department Length of Stay to Participants’ Characteristics

Supplementary Table 2. Differences in Emergency Department Revisit within 72 Hours to Participants’ Characteristics

## References

[1] Statistics Korea (2024). Cause of death statistics [Internet]. https://kosis.kr/statHtml/statHtml.do?orgId=101&tblId=DT_1B34E01&conn_path=I2.

[2] Statistics Korea (2024). Cancer registration statistics [Internet]. https://kosis.kr/statHtml/statHtml.do?orgId=117&tblId=DT_117N_A00023&conn_path=I2.

[3] Ministry of Health and Welfare (2024). Five-year relative survival rate by sex [Internet]. https://www.index.go.kr/unify/idx-info.do?idxCd=4241.

[4] Williams PA, Zaidi SK, Sengupta R (2024). AACR cancer progress report 2024: inspiring science-fueling progress-revolutionizing care. Clinical Cancer Research.

[5] Verginadis II, Citrin DE, Ky B, Feigenberg SJ, Georgakilas AG, Hill-Kayser CE (2025). Radiotherapy toxicities: mechanisms, management, and future directions. The Lancet.

[6] Lustberg MB, Kuderer NM, Desai A, Bergerot C, Lyman GH (2023). Mitigating long-term and delayed adverse events associated with cancer treatment: implications for survivorship. Nature Reviews Clinical Oncology.

[7] Tabriz AA, Turner K, Hong YR, Gheytasvand S, Powers BD, Lafata JE (2023). Trends and characteristics of potentially preventable emergency department visits among patients with cancer in the US. JAMA Network Open.

[8] Heo DS, Yoon YH, Jung JY, Kim HS, Kim SH, Shin SD (1998). Inappropriate care of oncologic emergency in Korea. Journal of Hospice and Palliative Care.

[9] Jung MS (2009). Research on the actual conditions of cancer patients in the emergency room in a university hospital in Seoul [dissertation].

[10] Rivera DR, Gallicchio L, Brown J, Liu B, Kyriacou DN, Shelburne N (2017). Trends in adult cancer-related emergency department utilization: an analysis of data from the nationwide emergency department sample. JAMA Oncology.

[11] Shin TG, Song JW, Song HG, Chong KH (2011). Characteristics of frequent users of emergency department. Journal of the Korean Society of Emergency Medicine.

[12] Yang SA, Cho OH, Yoo YS (2009). A survey of cancer patients who visited emergency room. Journal of Hospice and Palliative Care.

[13] Kim Y (2015). Healthcare policy and healthcare utilization behavior to improve hospital infection control after the Middle East respiratory syndrome outbreak. Journal of the Korean Medical Association.

[14] Han CY, Barnard A, Chapman H (2009). Emergency department nurses’ understanding and experiences of implementing discharge planning. Journal of Advanced Nursing.

[15] Sheikh H, Brezar A, Dzwonek A, Yau L, Calder LA (2018). Patient understanding of discharge instructions in the emergency department: do different patients need different approaches?. International Journal of Emergency Medicine.

[16] Ahmed AE, AlBuraikan DA, Almazroa HR, Alrajhi MN, ALMuqbil BI, Albaijan MA (2018). Seventy-two-hour emergency department revisits among adults with chronic diseases: a Saudi Arabian study. Therapeutics and Clinical Risk Management.

[17] El-Jawahri A, Nelson AM, Gray TF, Lee SJ, LeBlanc TW (2020). Palliative and end-of-life care for patients with hematologic malignancies. Journal of Clinical Oncology.

[18] Min HS, Chang HJ, Sung HK (2022). Emergency department utilization of adult cancer patient in Korea: a nationwide population-based study, 2017-2019. Cancer Research and Treatment.

[19] López-Aguilà S, Contel JC, Farré J, Campuzano JL, Rajmil L (2011). Predictive model for emergency hospital admission and 6-month readmission. The American Journal of Managed Care.

[20] Kim MH, Lee SJ, Choi MY, Kim DY, Yoo JS, Shin TG (2025). Factors that predict emergency department length of stay in analysis of national data. Clinical and Experimental Emergency Medicine.

[21] Shin YH, Chaung SK, Kim HJ (2019). Critical review I to standardize the textbooks of fundamentals of nursing: vital sign assessment, body temperature regulation, oxygenation. Journal of Korean Academy of Fundamentals of Nursing.

[22] West RM (2022). Best practice in statistics: the use of log transformation. Annals of Clinical Biochemistry.

[23] Ha BH, Park JY (2017). Triage and length of stay in a cancer center emergency department. Asian Oncology Nursing.

[24] Hsu J, Donnelly JP, Moore JX, Meneses K, Williams G, Wang HE (2018). National characteristics of emergency department visits by patients with cancer in the United States. The American Journal of Emergency Medicine.

[25] Lim SJ, Yi MS (2014). Study on cancer patients who visited an emergency department with the side effects of chemotherapy. Journal of Korean Clinical Nursing Research.

[26] Ohashi T, Takase-Minegishi K, Maeda A, Hamada N, Yoshimi R, Kirino Y (2023). Incidence and risk of hematological adverse events associated with immune checkpoint inhibitors: a systematic literature review and meta-analysis. Journal of Hematology.

[27] Wang L, Baser O, Kutikova L, Page JH, Barron R (2015). The impact of primary prophylaxis with granulocyte colony-stimulating factors on febrile neutropenia during chemotherapy: a systematic review and meta-analysis of randomized controlled trials. Supportive Care in Cancer.

[28] Scotté F, Taylor A, Davies A (2023). Supportive care: the “keystone” of modern oncology practice. Cancers.

[29] Lee SY, Ro YS, Shin SD, Moon SW (2021). Epidemiologic trends in cancer-related emergency department utilization in Korea from 2015 to 2019. Scientific Reports.

[30] National Cancer Information Center (2025). Cancer incidence by type [Internet]. https://www.cancer.go.kr/lay1/S1T639C641/contents.do.

[31] Son MH, Chung HS (2022). Chief complaints of patients with cancer who visit the emergency department over their oncologist’s outpatient clinic in South Korea. BMC Palliative Care.

[32] Evenepoel M, Haenen V, De Baerdemaecker T, Meeus M, Devoogdt N, Dams L (2022). Pain prevalence during cancer treatment: a systematic review and meta-analysis. Journal of Pain and Symptom Management.

[33] Goldberg EM, Wilson T, Saucier C, Brody AM, Levy PD, Eaton CB (2017). Achieving the BpTRUth: emergency department hypertension screening and the Centers for Medicare & Medicaid Services quality measure. Journal of the American Society of Hypertension.

[34] Strojnik K, Mahkovic-Hergouth K, Novakovic BJ, Seruga B (2016). Outcome of severe infections in afebrile neutropenic cancer patients. Radiology and Oncology.

[35] Ackroyd-Stolarz S, Guernsey JR, Mackinnon NJ, Kovacs G (2011). The association between a prolonged stay in the emergency department and adverse events in older patients admitted to hospital: a retrospective cohort study. BMJ Quality & Safety.

[36] Wong TH, Lau ZY, Ong WS, Tan KB, Wong YJ, Farid M (2018). Cancer patients as frequent attenders in emergency departments: a national cohort study. Cancer Medicine.

[37] Jones S, Moulton C, Swift S, Molyneux P, Black S, Mason N (2022). Association between delays to patient admission from the emergency department and all-cause 30-day mortality. Emergency Medicine Journal.

[38] Kochanek M, Schalk E, von Bergwelt-Baildon M, Beutel G, Buchheidt D, Hentrich M (2019). Management of sepsis in neutropenic cancer patients: 2018 guidelines from the Infectious Diseases Working Party (AGIHO) and Intensive Care Working Party (iCHOP) of the German Society of Hematology and Medical Oncology (DGHO). Annals of Hematology.

[39] Sokoloff C, Daoust R, Paquet J, Chauny JM (2014). Is adequate pain relief and time to analgesia associated with emergency department length of stay? A retrospective study. BMJ Open.

[40] Hughes JA, Brown NJ, Chiu J, Allwood B, Chu K (2020). The relationship between time to analgesic administration and emergency department length of stay: a retrospective review. Journal of Advanced Nursing.

[41] Shih TC, Chang HT, Lin MH, Chen CK, Chen TJ, Hwang SJ (2018). Differences in do-not-resuscitate orders, hospice care utilization, and late referral to hospice care between cancer and non-cancer decedents in a tertiary hospital in Taiwan between 2010 and 2015: a hospital-based observational study. BMC Palliative Care.

[42] Sung CW, Ho J, Fan CY, Chen CY, Chen CH, Lin SY (2024). Prediction of high-risk emergency department revisits from a machine-learning algorithm: a proof-of-concept study. BMJ Health & Care Informatics.

[43] Pellerin G, Gao K, Kaminsky L (2018). Predicting 72-hour emergency department revisits. The American Journal of Emergency Medicine.

[44] Blayney MC, Reed MJ, Masterson JA, Anand A, Bouamrane MM, Fleuriot J (2024). Multimorbidity and adverse outcomes following emergency department attendance: population based cohort study. BMJ Medicine.

[45] Jimenez-Puente A, del Rio-Mata J, Arjona-Huertas JL, Mora-Ordonez B, Nieto-de Haro L, Lara-Blanquer A (2015). Causes of 72-hour return visits to hospital emergency. Emergencias.

[46] Fruhan S, Bills CB (2022). Association of a callback program with emergency department revisit rates among patients seeking emergency care. JAMA Network Open.

