# Supplementary material for: Characteristics and clinical outcomes of cancer patients presenting to the emergency department in Korea: a retrospective descriptive study
Source: J Korean Biol Nurs Sci. 2026 Feb 23;28(1):206–16. [Article in Korean] doi: 10.7586/jkbns.25.077 (PMC13268344; doi:10.7586/jkbns.25.077)
Supplement: Supplementary file 1 [file jkbns-25-077-Supplementary-Table-1.pdf]

Supplementary Table 1. Differences in Emergency Department Length of Stay to Participants' Characteristics (N = 2,225)

| Characteristics | | ED LOS | | Log-transformed ED LOS | | |
|---|---|---|---|---|---|---|
| | | n | M±SD (min) | M±SD | t/F | p (post-hoc) |
| *General characteristics* | | | | | | |
| Sex | Men | 1,135 | 247.78±189.86 | 5.23±0.82 | 0.35 | .723 |
| | Women | 1,090 | 244.01±189.79 | 5.22±0.80 | | |
| Age (years) | ≤ 49$^{a}$ | 378 | 228.52±158.97 | 5.19±0.75 | 5.99 | <.001 |
| | 50 ~ 59$^{b}$ | 359 | 241.64±206.14 | 5.18±0.81 | | (e> |
| | 60 ~ 69$^{c}$ | 605 | 238.40±181.09 | 5.19±0.83 | | a,b,c,d) |
| | 70 ~ 79$^{d}$ | 499 | 234.77±173.20 | 5.19±0.81 | | |
| | ≥ 80$^{e}$ | 384 | 293.45±225.86 | 5.41±0.80 | | |
| Residence | Metropolitan | 1,753 | 248.33±190.71 | 5.25±0.78 | 2.27 | .023 |
| | Non-metropolitan | 472 | 237.01±186.27 | 5.15±0.89 | | |
| Prior ED visit | First visit | 652 | 222.92±181.71 | 5.12±0.81 | -4.08 | <.001 |
| | ≥1 prior visits | 1,573 | 255.47±192.29 | 5.27±0.81 | | |
| Prior hospitalization | Yes | 1,311 | 251.63±194.52 | 5.25±0.82 | 1.63 | .103 |
| (within 6 months) | No | 914 | 237.76±182.59 | 5.19±0.80 | | |
| Comorbidities | | | | | | |
| Hypertension | Yes | 672 | 261.67±200.39 | 5.28±0.82 | 2.17 | .031 |
| | No | 1,553 | 239.12±184.67 | 5.20±0.80 | | |
| Diabetic mellitus | Yes | 426 | 279.75±207.64 | 5.35±0.83 | 3.47 | .001 |
| | No | 1,799 | 237.93±184.47 | 5.20±0.80 | | |
| *Cancer-related characteristics* | | | | | | |
| Cancer diagnosis | | | | | | |
| Gastrointestinal | Yes | 1,103 | 251.86±176.69 | 5.26±0.81 | 2.14 | .032 |
| | No | 1,122 | 240.11±201.75 | 5.19±0.80 | | |
| Genitourinary | Yes | 387 | 257.07±221.39 | 5.24±0.84 | 0.48 | .632 |
| /gynecologic | No | 1,838 | 243.59±182.42 | 5.22±0.80 | | |
| Head and neck | Yes | 370 | 238.22±212.52 | 5.17±0.79 | -1.46 | .144 |
| | No | 1,855 | 247.47±184.95 | 5.24±0.81 | | |
| Breast | Yes | 282 | 223.83±195.10 | 5.12±0.80 | -2.35 | .019 |
| | No | 1,943 | 249.14±188.84 | 5.24±0.81 | | |
| Lung | Yes | 172 | 263.44±202.33 | 5.35±0.72 | 2.27 | .024 |
| | No | 2,053 | 244.47±188.68 | 5.22±0.81 | | |
| Metastasis | Yes | 690 | 254.97±209.76 | 5.23±0.86 | 0.27 | .791 |
| | No | 1,535 | 241.87±180.02 | 5.22±0.78 | | |
| DNR status | Yes | 631 | 271.09±199.63 | 5.32±0.84 | 3.50 | <.001 |
| | No | 1,594 | 235.97±184.87 | 5.19±0.79 | | |
| Chemotherapy | Yes | 1,196 | 251.31±187.93 | 5.26±0.81 | 1.98 | .048 |
| | No | 1,029 | 239.68±191.83 | 5.19±0.81 | | |
| Radiotherapy | Yes | 817 | 246.32±187.91 | 5.23±0.83 | 0.07 | .947 |
| | No | 1,408 | 245.71±190.94 | 5.23±0.80 | | |
| Surgery | Yes | 1,505 | 243.90±193.69 | 5.22±0.80 | -0.69 | .490 |
| | No | 720 | 250.18±181.42 | 5.24±0.82 | | |
| *ED visit-related characteristics* | | | | | | |
| Arrival time | Business hours | 1,353 | 235.28±178.28 | 5.18±0.82 | -3.29 | .001 |
| | Off-hours | 872 | 262.46±205.40 | 5.30±0.78 | | |
| Acuity | Emergent | 1,541 | 267.92±197.38 | 5.34±0.77 | 9.92 | <.001 |

| Characteristics | | ED LOS | | Log-transformed ED LOS | | |
|---|---|---|---|---|---|---|
| | | n | M±SD (min) | M±SD | t/F | *p* (post-hoc) |
| Mode of arrival | Non-emergent | 684 | 196.40±160.95 | 4.97±0.83 | -9.00 | <.001 |
| | Walk-in | 1,791 | 230.88±178.02 | 5.16±0.82 | | |
| | Ambulance | 434 | 308.06±221.79 | 5.51±0.70 | | |
| Chief complaint | | | | | | |
| Abdominal pain | Yes | 388 | 252.84±148.84 | 5.36±0.64 | 4.12 | <.001 |
| | No | 1,837 | 244.47±197.36 | 5.20±0.84 | | |
| Fever | Yes | 301 | 272.35±161.14 | 5.42±0.67 | 5.30 | <.001 |
| | No | 1,924 | 241.80±193.60 | 5.20±0.82 | | |
| Dyspnea | Yes | 171 | 289.75±215.37 | 5.35±0.93 | 2.07 | .039 |
| | No | 2,054 | 242.28±187.10 | 5.22±0.80 | | |
| General weakness | Yes | 131 | 248.20±177.93 | 5.19±0.92 | -0.47 | .642 |
| | No | 2,094 | 245.79±190.55 | 5.23±0.80 | | |
| Nausea/vomiting | Yes | 66 | 274.55±193.53 | 5.39±0.72 | 1.71 | .087 |
| | No | 2,159 | 245.06±189.65 | 5.22±0.81 | | |
| Pulse rate (/min) | Normal (60 ~ 100) | 1,529 | 237.36±192.08 | 5.19±0.80 | -3.44 | .001 |
| | Abnormal range (< 60 and > 100) | 696 | 264.77±183.40 | 5.31±0.82 | | |
| Body temperature (℃) | Normal (36.1 ~ 37.2) | 1,515 | 238.86±197.22 | 5.18±0.83 | -4.38 | <.001 |
| | Abnormal range (< 36.1 and >37.2) | 710 | 261.03±172.02 | 5.33±0.75 | | |
| Systolic blood pressure(mmHg) | Normal (90 ~ 119) | 549 | 243.16±189.74 | 5.17±0.89 | -1.64 | .102 |
| | Abnormal range (< 90 and ≥ 120) | 1,676 | 246.84±189.86 | 5.24±0.78 | | |
| Oxygen saturation (%) | Normal (95 ~ 100) | 2,112 | 243.03±188.77 | 5.22±0.80 | -2.70 | .007 |
| | Abnormal range (< 95) | 113 | 300.11±201.34 | 5.43±0.89 | | |
| Respiratory rate (/min) | Normal (12 ~ 20) | 2,163 | 242.27±186.00 | 5.21±0.81 | -4.84 | <.001 |
| | Abnormal range (< 12 and > 20) | 62 | 373.65±264.60 | 5.71±0.70 | | |
| NSAIDs | Yes | 113 | 254.50±154.75 | 5.38±0.59 | 2.76 | .007 |
| | No | 2,112 | 245.47±191.50 | 5.22±0.82 | | |
| Acetaminophen | Yes | 461 | 301.47±190.94 | 5.54±0.58 | 11.75 | <.001 |
| | No | 1,764 | 231.42±186.84 | 5.14±0.84 | | |
| Opioid | Yes | 298 | 301.73±229.53 | 5.49±0.67 | 7.11 | <.001 |
| | No | 1,927 | 237.30±181.42 | 5.19±0.82 | | |
| Antiemetics | Yes | 248 | 263.23±195.85 | 5.34±0.67 | 2.81 | .005 |
| | No | 1,977 | 243.76±188.96 | 5.21±0.82 | | |
| Oxygen therapy in the ED | Yes | 125 | 317.81±248.51 | 5.46±0.86 | 3.34 | .001 |
| | No | 2,100 | 241.65±184.91 | 5.21±0.80 | | |
| ED disposition | Admission | 1,243 | 270.28±202.39 | 5.30±0.88 | 4.96 | <.001 |
| | Discharge | 982 | 215.11±167.62 | 5.13±0.70 | | |

DNR = Do-not-resuscitation; ED = Emergency department; LOS = Length of stay; M = Mean; ; NSAIDs = Non-steroidal antiinflammatory drug; SD = Standard deviation.
