# Supplementary material for: Characteristics and clinical outcomes of cancer patients presenting to the emergency department in Korea: a retrospective descriptive study
Source: J Korean Biol Nurs Sci. 2026 Feb 23;28(1):206–16. [Article in Korean] doi: 10.7586/jkbns.25.077 (PMC13268344; doi:10.7586/jkbns.25.077)
Supplement: Supplementary file 2 [file jkbns-25-077-Supplementary-Table-2.pdf]

Supplementary Table 2. Differences in Emergency Department Revisit within 72 Hours to Participants' Characteristics (N = 2,225)

Characteristics		ED revisit within 72 hours Yes (n = 121) n (%)	No (n = 2,104) n (%)	χ^2	*p*
General characteristics					
Sex	Men	59(5.2)	1,076(94.8)	0.26	.611
	Women	62(5.7)	1,028(94.3)		
Age (years)	≤ 49	20(5.3)	358(94.7)	2.07	.724
	50 ~ 59	16(4.5)	343(95.5)		
	60 ~ 69	33(5.5)	572(94.5)		
	70 ~ 79	26(5.2)	473(94.8)		
	≥ 80	26(6.8)	358(93.2)		
Residence	Metropolitan	103(5.9)	1,650(94.1)	3.08	.080
	Non-metropolitan	18(3.8)	454(96.2)		
Prior ED visit	First visit	28(4.3)	624(95.7)	2.35	.126
	≥1 prior visits	93(5.9)	1,480(94.1)		
Prior hospitalization (within 6 months)	Yes	82(6.3)	1,229(93.7)	4.14	.042
	No	39(4.3)	875(95.7)		
Comorbidities					
Hypertension	Yes	45(6.7)	627(93.3)	2.96	.085
	No	76(4.9)	1,477(95.1)		
Diabetic mellitus	Yes	24(5.6)	402(94.4)	0.04	.843
	No	97(5.4)	1,702(94.6)		
Cancer-related characteristics					
Cancer diagnosis					
Gastrointestinal	Yes	50(4.5)	1,053(95.5)	3.49	.062
	No	71(6.3)	1,051(93.7)		
Genitourinary /gynecologic	Yes	24(6.2)	363(93.8)	0.53	.466
	No	97(5.3)	1,741(94.7)		
Head and neck	Yes	25(6.8)	345(93.2)	1.50	.221
	No	96(5.2)	1,759(94.8)		
Breast	Yes	11(3.9)	271(96.1)	1.48	.223
	No	110(5.7)	1,833(94.3)		
Lung	Yes	13(7.6)	159(92.4)	1.63	.202
	No	108(5.3)	1,945(94.7)		
Metastasis	Yes	39(5.7)	651(94.3)	0.09	.765
	No	82(5.3)	1,453(94.7)		
DNR status	Yes	53(8.4)	578(91.6)	15.02	<.001
	No	68(4.3)	1,526(95.7)		
Chemotherapy	Yes	68(5.7)	1,128(94.3)	0.31	.579
	No	53(5.2)	976(94.8)		
Radiotherapy	Yes	40(4.9)	777(95.1)	0.74	.390
	No	81(5.8)	1,327(94.2)		
Surgery	Yes	88(5.8)	1,417(94.2)	1.51	.219
	No	33(4.6)	687(95.4)		
ED visit-related characteristics					
Arrival time	Business hours	62(4.6)	1,291(95.4)	4.92	.027
	Off-hours	59(6.8)	813(93.2)		
Acuity	Emergent	91(5.9)	1,450(94.1)	2.13	.145

Characteristics		ED revisit within 72 hours Yes (n = 121)	No (n = 2,104)	χ²	*p*
		n (%)			
Mode of arrival	Non-emergent	30(4.4)	654(95.6)	0.72	.395
	Walk-in	101(5.6)	1,690(94.4)		
	Ambulance	20(4.6)	414(95.4)		
Chief complaint					
Abdominal pain	Yes	25(6.4)	363(93.6)	0.92	.337
	No	96(5.2)	1,741(94.8)		
Fever	Yes	18(6.0)	283(94.0)	0.20	.656
	No	103(5.4)	1,821(94.6)		
Dyspnea	Yes	13(7.6)	158(92.4)	1.69	.194
	No	108(5.3)	1,946(94.7)		
General weakness	Yes	8(6.1)	123(93.9)	0.12	.728
	No	113(5.4)	1,981(94.6)		
Nausea/vomiting	Yes	4(6.1)	62(93.9)	0.05	.821
	No	117(5.4)	2,042(94.6)		
Pulse rate (/min)	Normal (60 ~ 100)	81(5.3)	1,448(94.7)	0.19	.665
	Abnormal range (< 60 and > 100)	40(5.7)	656(94.3)		
Body temperature (℃)	Normal (36.1 ~ 37.2)	83(5.5)	1,432(94.5)	0.02	.902
	Abnormal range (< 36.1 and >37.2)	38(5.4)	672(94.6)		
Systolic blood pressure(mmHg)	Normal (90 ~ 119)	40(7.3)	509(92.7)	4.84	.028
	Abnormal range (< 90 and ≥ 120)	81(4.8)	1,595(95.2)		
Oxygen saturation (%)	Normal (95 ~ 100)	116(5.5)	1,996(94.5)	0.24	.626
	Abnormal range (< 95)	5(4.4)	108(95.6)		
Respiratory rate (/min)	Normal (12 ~ 20)	117(5.4)	2,046(94.6)	0.13	.721
	Abnormal range (< 12 and > 20)	4(6.5)	58(93.5)		
NSAIDs	Yes	10(8.8)	103(91.2)	2.69	.101
	No	111(5.3)	2,001(94.7)		
Acetaminophen	Yes	25(5.4)	436(94.6)	0.00	.987
	No	96(5.4)	1,668(94.6)		
Opioid	Yes	23(7.7)	275(92.3)	3.48	.062
	No	98(5.1)	1,829(94.9)		
Antiemetics	Yes	17(6.9)	231(93.1)	1.09	.297
	No	104(5.3)	1,873(94.7)		
Oxygen therapy in the ED	Yes	7(5.6)	118(94.4)	0.01	.935
	No	114(5.4)	1,986(94.6)		
ED disposition	Admission	49(3.9)	1,194(96.1)	12.26	<.001
	Discharge	72(7.3)	910(92.7)		

DNR = Do-not-resuscitation; ED = Emergency department; NSAIDs = Non-steroidal anti inflammatory drug.
